# Early and subsequent radiographic changes during the occurrence of osteochondritis dissecans of the elbow

**DOI:** 10.1016/j.jseint.2025.03.015

**Published:** 2025-04-15

**Authors:** Masatoshi Takahara, Tomohiro Uno, Tamotsu Kamishima, Daiichiro Takahara, Ryo Mitachi, Hiroshi Satake, Michiaki Takagi

**Affiliations:** a Center for Hand, Elbow, and Sports Medicine, Izumi Orthopaedic Hospital, Sendai, Japan; b Department of Orthopaedic Surgery, Yamagata University Faculty of Medicine, Yamagata, Japan; c Faculty of Health Sciences, Hokkaido University, Sapporo, Japan

**Keywords:** Osteochondritis dissecans, Elbow, Baseball, Diagnosis, Subchondral bone, Cyst, Stress injury, Physis

## Abstract

**Background:**

Little is known about the radiographic changes before and after osteochondritis dissecans (OCD) occurrence. The aim was to clarify the earliest and subsequent radiographic changes.

**Methods:**

Among 120 patients with capitellar OCD, we selected the patients who had consecutive radiographs of the elbow before and after OCD occurrence. We retrospectively clarified the earliest and subsequent changes.

**Results:**

Four (3%) boys met the criteria. All four had been in baseball team and had medial elbow pain with medial epicondylar apophysitis. They had no lateral elbow pain or abnormal findings of the capitellum. After the mean of 3.5 months from initial presentation, OCD silently occurred in the capitellum at the mean age of 11.4 years. The earliest detectable radiographic change was subtle rarefaction at the ossifying subchondral bone surface in the lateral aspect of the capitellum; however, it was too subtle and limited to be recognizable as OCD. These features gradually became more evident and expanded, eventually leading to flattening and depression of the subchondral bone surface, accompanied by subchondral bone cysts surrounded by sclerotic bone. The capitellar lesions were diagnosed as early OCD.

**Discussion:**

This is the first report to show the radiographic changes before and after OCD occurrence. The earliest change was subtle rarefaction. Repetitive forces on the preadolescent capitellum may cause stress injury at the secondary physis followed by rarefaction of the ossifying subchondral bone. Micromovements of the overlying cartilage would allow the side-by-side concurrence of subchondral bone cysts, resulting in subchondral depression, eventually leading to recognizable early OCD.

Osteochondritis dissecans (OCD) is a disorder of the joint that may cause progressive changes in the cartilage and subchondral bone, including softening, swelling, early separation, partial detachment, and complete osteochondral separation with a loose body.^32^^,^^41^ This condition was described as “quiet necrosis” by Sir James Paget in 1870 and later termed “osteochondritis dissecans” by König in 1888, who proposed that an inflammatory reaction in the bone and articular cartilage caused spontaneous necrosis.^21^ For over 150 years, many etiological theories have been proposed for OCD, including genetic predisposition, ischemia, and repetitive or acute trauma.^2^^,^^6^^,^^14^^,^^20^^,^^21^^,^^24^^,^^25^^,^^29^^,^^33^, ^34^, ^35^^,^^38^^,^^40^^,^^43^^,^^44^^,^^49^^,^^51^ However, its etiology has remained unclear. Trauma, either acute or repetitive microtrauma, has become a more accepted cause of OCD due to the high incidence of this disorder in athletes.^6^^,^^14^^,^^23^^,^^26^^,^^42^^,^^52^

Histologically, OCD lesions are divided into the following three layers: 1) an articular fragment (progeny fragment), 2) an intermediate layer (gap), and 3) the underlying bone (parent bone).^4^^,^^22^^,^^42^^,^^51^ These three layers of OCD can begin with separation caused by compressive and shear forces to the immature capitellum.^42^ Osteonecrosis has been observed in less than half of the articular fragments of the specimens.^22^^,^^42^^,^^48^^,^^51^^,^^52^ In contrast, the viability of the underlying bone is nearly normal in all cases.^22^^,^^42^ The pathologic progression of the articular fragments in OCD has been reported to begin with separation beneath the immature epiphyseal cartilage (IA), followed by ossification arrest (IB) or delay (IIA), and finally osteonecrosis (IIB) as a late event.^48^ Patients with early OCD are usually at stage IA and younger, with a lower skeletal age and shorter duration of symptoms.^48^ These findings suggest that OCD may occur in the preadolescent capitellum and gradually change the appearance, eventually being diagnosed at a variety of stages as well as durations from the occurrence.

An anteroposterior radiograph of the elbow at 45° of flexion (APR45) has been suggested to increase the diagnostic accuracy of capitellar OCD.^28^^,^^36^^,^^41^^,^^43^^,^^44^ APR45 can detect slight changes in early OCD while standard radiographs (AP and lateral views) fail to show abnormalities.^28^^,^^36^^,^^41^^,^^44^ Early OCD lesions at stage I are characterized by slight changes to the subchondral bone of the humeral capitellum before epiphyseal closure, such as radiolucency, flattening, and depression.^8^^,^^12^^,^^13^^,^^16^^,^^18^^,^^27^^,^^28^^,^^37^^,^^44^^,^^45^ OCD lesions with an intact articular cartilage are stable and have the potential to heal spontaneously.^13^^,^^15^^,^^18^^,^^28^^,^^46^^,^^50^ As the surgical intervention is not indicated for stable OCD lesions at stage I, little is known about the early pathologic changes that occur before and after OCD lesions become recognizable. Therefore, it would be useful to investigate radiographic changes shortly after early OCD lesions appear. However, we have been unaware of any reports that showed the radiographic changes before and after OCD occurrence. During the observation periods of elbow disorders except for OCD, we had rare opportunities to obtain consecutive radiographs before and after OCD occurrence. The aim of this study was to clarify the earliest and subsequent radiographic changes associated with OCD occurrence.

## Materials and methods

This was a retrospective, single-center diagnostic study approved by our institutional review board.

Between April 2015 and December 2019, we treated 120 patients aged ≤20 years with OCD of the humeral capitellum at Izumi Orthopaedic Hospital. There were 117 males and 3 females, including 99 patients who had been reported previously.^42^^,^^47^^,^^48^ All patients had belonged to sports clubs and played competitively. The sport was baseball in 108 cases, tennis in 4, shot-put in 2, gymnastics in 2, basketball in 1, badminton in 1, dodgeball in 1, and skateboarding in 1. The mean age at the start of treatment was 13.7 years (range, 9.0-20.3 years), and the mean skeletal age score for the elbow using the Sauvegrain method (0-27 points system)^5^ was 22.9 points (range, 7-27 points).

We set the selection criteria for the patients: 1) There were radiographs including APR45 before OCD occurrence, 2) There were consecutive radiographs showing the changes from the intact to OCD, and 3) There were no episodes of fracture or dislocation involving the elbow. We retrospectively reviewed consecutive radiographs of the elbow during OCD occurrence to clarify the earliest and subsequent radiographic changes.

## Results

**Figure 1:**
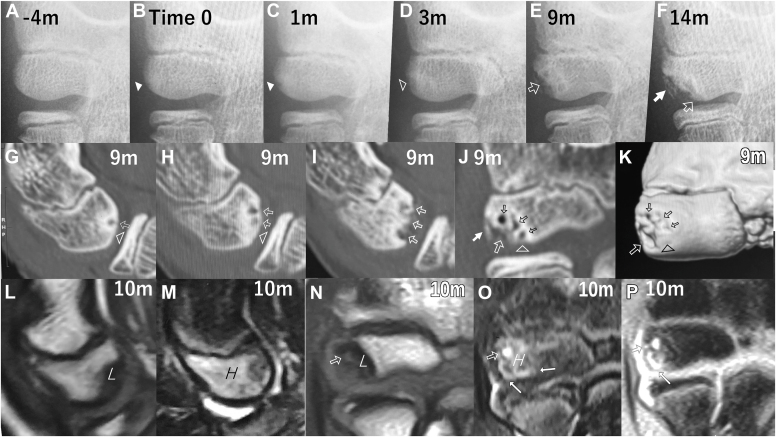
Case 1, a 10.5-year-old boy with osteochondritis dissecans of the capitellum. (**A**-**F**) Anteroposterior radiographs of the elbow at 45° of flexion (APR45). (**A**) APR45 at initial presentation showing no abnormal findings of the capitellum. (**B**) APR45, taken 4 months after initial presentation, showing the first detectable feature that was extremely subtle rarefaction surrounded by sclerosis within a limited area of the ossifying subchondral bone surface in the lateral aspect of the capitellum (*arrowhead*). This time was assumed to be Time 0. (**C**, **D**) APR45, taken 1 and 3 months after Time 0, showing progression from the rarefaction (*arrowhead*) to flattening (*open arrowhead*). (**E**) APR45, taken 9 months after Time 0, showing subchondral bone depression (*open arrow*) and an uneven bony floor surrounded by osteosclerosis. He was diagnosed to have OCD at radiographic stage I and was treated nonoperatively with activity restriction. (**F**) APR45, taken 14 months after Time 0, showing subchondral depression expanding to the central aspect of the capitellum (*open arrow*) and endochondral ossification (*white arrow*) along the original capitellar bone surface in the lateral. (**G**-**K**) CT images taken 9 months after Time 0. (**L**-**P**) MRI taken 10 months after Time 0. (**G**, **H**, **I**) Sagittal CT images, reconstructed along a plane with 10° in valgus,^41^ showing flattening (*open arrowheads*), small subchondral bone cysts (**G**, **H**; *open arrows*), and depression (**I**, *open arrows*) surrounded by osteosclerosis. (**J**, **K**) Coronal CT image, reconstructed along a plane with a tilting angle of 55°,^41^ and 3D-CT image showing flattening (*open arrowhead*), a partial bone defect (*large open arrow*), small cyst-like depressions (*small open arrows*) surrounded by osteosclerosis, and ossification in the lateral (**J**, *white arrow*). (**L**, **M**) Sagittal MRI taken along a plane with 10° in valgus.^41^ (**N**, **O**, **P**) Coronal MRI taken along a plane with a tilting angle of 55°.^41^ (**L**, **N**) T1-weighted MRI showing subchondral bone cyst (*arrow*) and low signal area (*L*) consistent with osteogenic changes and bone defect. (**M**, **O**, **P**) Fat-suppressed T2-and T2∗-weighted MRI showing a high-signal interface (*white arrow*), a subchondral bone cyst (*open arrow*), and high signal area (*H*) consistent with bone marrow edematous changes. *3D*, three dimensional; *CT*, computed tomography.

**Figure 2:**
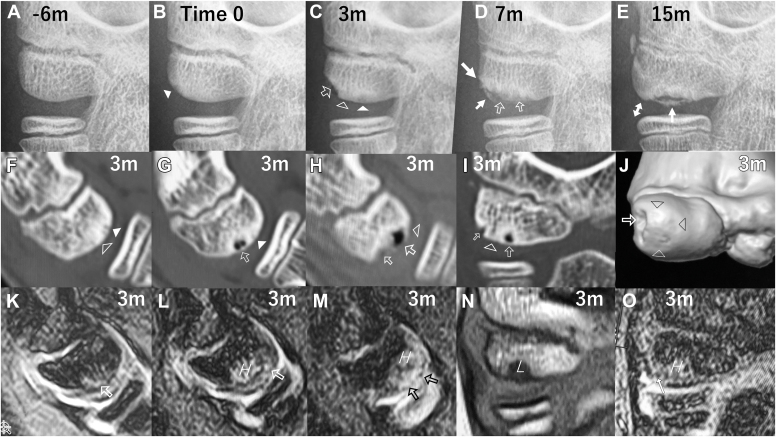
Case 2, a 11.7-year-old boy with osteochondritis dissecans of the capitellum. (**A**-**E**) Anteroposterior radiographs of the elbow at 45° of flexion (APR45). (**A**) APR45 at initial presentation showing no abnormal findings of the capitellum. (**B**) APR45, taken 6 months after initial presentation, showing the first detectable feature that was extremely subtle rarefaction surrounded by sclerosis with a limited area of the ossifying subchondral bone surface in the lateral aspect of the capitellum (*arrowhead*). This time was assumed to be Time 0. (**C**) APR45, taken 3 months after Time 0, showing subchondral bone rarefaction (*arrowhead*), flattening (*open arrowhead*), and depression (*open arrow*) surrounded by osteosclerosis. He was diagnosed to have OCD and treated nonoperatively. (**D**) APR45, taken 7 months after Time 0, showing enlargement of subchondral bone depression (*open arrows*) surrounded by uneven osteosclerosis in the central aspect of the capitellum and 2-way ossification from the underlying bone (*large white arrow*) and along the original capitellar bone surface (*small white arrow*). (**E**) APR45, taken 15 months after Time 0, showing union in the lateral aspect of the capitellum (*double arrow*) and nonunion in the central aspect (*arrow*). (**F**-**O**) CT images and MRI taken 3 months after Time 0. (**F**, **G**, **H**, **I**) Sagittal CT images, reconstructed along a plane with 10° in valgus,^41^ and coronal CT image, reconstructed along a plane with a tilting angle of 55°,^41^ showing flattening (**F**, **H**, **I**: *open arrowhead*) and subchondral bone cysts (**G**, **H**, **I**: *open arrows*) surrounded by osteosclerosis. (**J**) 3D-CT image showing a partial bone defect (*open arrow*) and flattening (*arrowheads*). (**K**, **L**, **M**) Sagittal MRI taken along a plane with 10° in valgus.^41^ (**N**, **O**) Coronal MRI taken along a plane with a tilting angle of 55°.^41^ (**K**, **L**, **M**, **O**) Fat-suppressed T2-and T2∗-weighted MRI showing focal high signal lesions (**K**, **L**, **M**, *open arrows*), a high-signal interface (**O**, *white arrow*), and high signal area (*H*) consistent with bone effusion. (**N**) T1-weighted MRI showing low signal area (*L*) consistent with osteogenic changes and bone defect. *CT*, computed tomography; *MRI*, magnetic resonance imaging.

Among the 120 patients, only four (3%) met the above criteria (Table I). All four boys had been in baseball team (4 pitchers, 2 fielders, and 1 catcher, including 3 overlaps). At initial presentation, they had medial elbow pain for a mean of 4.5 months (range, 1-10 months) and were diagnosed to have medial epicondylar apophysitis (MEA). They had no lateral elbow pain or abnormal radiographic features of the capitellum (Figs. 1, *A* and 2, *A*). After 1 month of elbow rest, they returned to baseball without elbow pain.

**Table I:** Selected patients with consecutive radiographs during the occurrence of osteochondritis dissecans (OCD) of the capitellum.

Case	Sports baseball	Initial presentaion (IP)			Capitellar OCD occurrence					Capitellar OCD diagnosis		
		Age (yr)	Elbow pain location	Diganosis APR45	Period from IP (mo)	Age (yr)	Skeletal age (points)		Elbow pain	Period from IP (mo)	Age (yr)	Elbow pain
							Elbow	Capitellum				
1	P/F	10.1	Medial	MEA	4	10.4	14	3	None	13	11.2	None
2	P/F	11.1	Medial	MEA	6	11.7	8	3	None	9	11.9	None
3	P	11.4	Medial	MEA	3	11.6	9	3	None	5	11.8	None
4	P/C	11.9	Medial	MEA	1	12	12	3	None	1	12	None
Mean		11.1			3.5	11.4	10.8	3		7	11.7	

*OCD*, osteochondraotis dissecans; *MEA*, medial epicondylar apophysitis; *P*, pitcher; *F*, fielder; *C*, catcher; *APR45*, anteroposterior radiographs of the elbow at 45 degrees of flexion.

Compared to the prior radiographs, APR45, taken 1-6 months (mean, 3.5 months) after initial presentation, showed the earliest detectable change, ie, extremely subtle rarefaction surrounded by sclerosis. At that time, the mean patient's age was 11.4 years (range, 10.4-12.0 years) with a mean skeletal age score of 10.8 points for the elbow (0-27 points system) and 3 points for the capitellum (0-9 points system). The earliest detectable change occurred within a limited area of the ossifying subchondral bone surface in the lateral aspect of the capitellum (Figs. 1, *B*, and 2, *B*), and this was not diagnosed as OCD at that time. The early changes gradually became more evident and expanded, eventually leading to flattening and depression of the subchondral bone surface (Figs. 1, *C-E*, and 2, *C*).

At 1-13 months (mean, 7 months) after initial presentation, ie, 0-9 months (mean, 3.5 months) after the appearance of the earliest change, the capitellar lesions were diagnosed as asymptomatic OCD at radiographic stage I^41^ (Figs. 1, *E*, and 2, *C*). Magnetic resonance imaging (MRI) and computed tomography (CT) scans were taken within 1 month after OCD diagnosis, except for 1 case (case 4). Compared to APR45 showing subchondral bone flattening and depression, CT scan more clearly showed the changes, including i) small subchondral bone cysts covered by flattened subchondral bone, which was thin, discontinuous or absent in places (Figs. 1, *G-K*, and 2, *F-J*), ii) many small depressions at the floor of a partial bone defect (Figs. 1, *I-K*, and 2, *H-J*), and iii) osteosclerotic changes surrounding subchondral bone cysts and depressions (Figs. 1, *G-J*, and 2, *F-I*). MRI clearly revealed the changes, such as i) a T1-low signal area consistent with a partial bone defect and surrounding osteosclerosis (Figs. 1, *L*, *N*, and 2, *N*), ii) a T2-high-signal interface around the subchondral bone surface (Figs. 1, *O*, *P*, and 2, *O*), iii) a T2-high-signal area or a focal subchondral bone cyst at the site of the bone defect (Figs. 1, *O*, *P*, and 2, *K-M*), and iv) edematous changes in the marrow (Figs. 1, *M*, *O*, and 2, *L*, *M*, *O*). There was no evidence of articular cartilage fracture or displacement of the articular fragment. These lesions were assessed as stable OCD at stage IA.

All four cases were treated nonoperatively with activity restriction. Nevertheless, APR45, taken within 3 months after the start of treatment, showed that subchondral bone flattening and depression expanded to the central aspect of the capitellum (Figs. 1, *F*, and 2, *D*). Case 3 dropped out after observation for 4 months. Within 9 months, 2-way ossification appeared along the outline of the original subchondral bone surface and from the floor of the underlying bone (Figs. 1, *F*, and 2, *D-E*), eventually resulting in complete healing (cases 1 and 4) or a nonunited osteochondral fragment (case 2). These pathologic and healing changes occurred on the lateral aspect of the capitellum, followed by the central aspect (Figs. 1 and 2).

## Discussion

The retrospective radiographic review revealed that APR45 showed subtle features suggestive of OCD, ie, rarefaction surrounded by sclerosis within a limited area of the ossifying subchondral bone surface in the lateral aspect of the capitellum (Figs. 1, *B*, and 2, *B*). These findings were too subtle to have been diagnosed as OCD at the time. The changes that occurred subsequently were flattening and depression of the ossifying subchondral bone surface, diagnosed as radiographic stage I OCD (Figs. 1, *E*, and 2, *C*). These depressive changes were accompanied by subchondral bone cysts and an uneven bony floor of the depression surrounded by osteosclerotic changes (Figs. 1, *G-K*, and 2, *F-J*). This study was able to demonstrate that OCD can appear silently in the form of subtle rarefaction surrounded by sclerosis within a limited area of the ossifying subchondral bone surface in the lateral aspect of the capitellum. This is the first report to show the earliest and subsequent changes associated with OCD occurrence. The earliest detectable radiographic features are very interesting.

**Figure 3:**
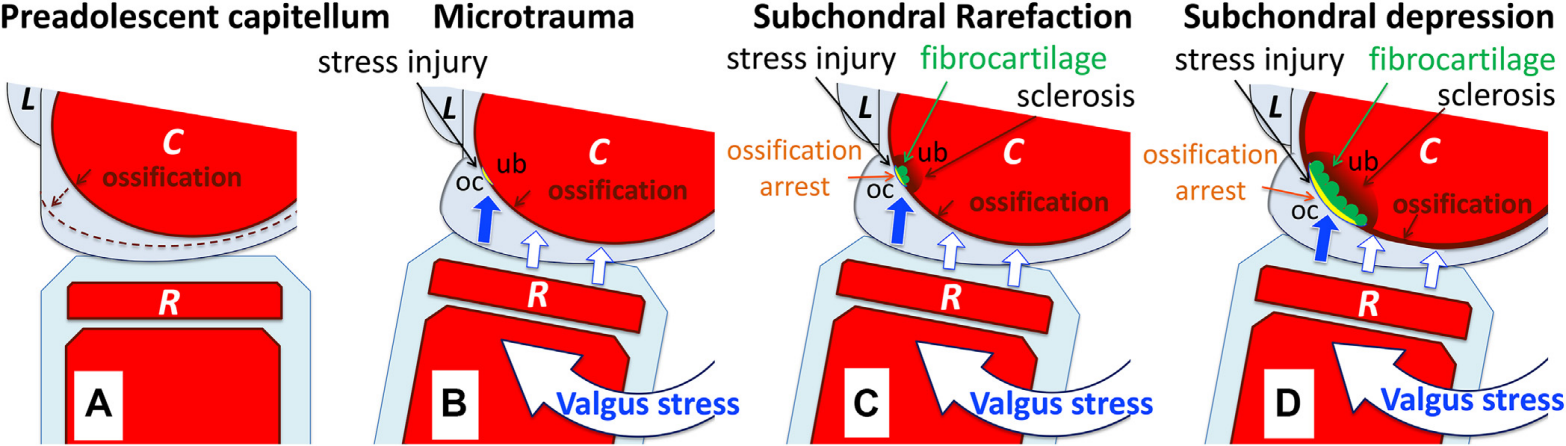
Early pathology in osteochondritis dissecans (OCD) of the capitellum in preadolescent baseball players. Illustrations showing the anterior aspect (**A**-**D**). (**A**) When the ossifying subchondral bone surface (*brown arc*) in the lateral aspect of the capitellum (*C*) is still spherical in shape, the overlying cartilage remains thicker laterally. Dotted line suggesting a future outline of the matured subchondral bone. (**B**-**D**) During repetitive pitched, compression (*white arrows*) and shear (*blue arrows*) forces to the preadolescent capitellum (*C*) from the radial head (*R*) may mainly act as shear forces (**B**-**D**: *blue arrows*) on the spherical subchondral bone surface in the lateral aspect of the capitellum, and accelerated elbow extension increases the shear forces. (**B**) Increased shear forces may cause repetitive microtrauma (stress injury: *yellow arc*) at the secondary physis between the overlying cartilage (*oc*) and underlying bone (*ub*), followed by subchondral rarefaction within a limited area in the lateral aspect of the capitellum. (**C**) Secondary physeal stress injury (*yellow arc*) may be followed by ossification arrest, fibrocartilage covering on the injured bone surface, and reactive osteosclerosis of the underlying bone (*ub*). (**D**) Repetitive micromovements of the overlying cartilage (*oc*) may allow expansion of stress injury and side-by side concurrence of subchondral bone cysts, leading to subchondral bone depression with an uneven floor surrounded by reactive osteosclerosis. Overlying cartilage (*oc*). Underlying bone (*ub*). Subchondral bone cyst (**C**, **D**: *green round*).

All four patients actively played baseball and had medial elbow pain caused by MEA. During throwing, the elbow is exposed to repetitive valgus and extension stress, resulting in distraction forces to the ulnar collateral ligament, accompanied by compressive and shear forces on the capitellum (Fig. 3, *A*, *B*). The most accepted cause of OCD is repetitive microtrauma.^3^^,^^6^^,^^14^^,^^23^^,^^26^^,^^42^^,^^52^ The present findings suggest that repetitive forces on the preadolescent capitellum cause microtrauma, leading to subchondral bone rarefaction surrounded by sclerosis, eventually resulting in OCD (Fig. 3, *B*).

Other than MEA and OCD, overuse injuries in preadolescent baseball players frequently involve the medial epicondylar apophysis, olecranon apophysis, and proximal humeral physis, leading to so-called Little Leaguer Elbow^1^^,^^11^^,^^39^ and Shoulder.^1^^,^^10^^,^^31^ These stress injuries at the primary physis (primary physeal stress injuries [primary PSIs]) show characteristic changes to the physis, including ossification arrest, widening, delayed closure, and acute or stress fracture, accompanied by the adjacent metaphyseal bone rarefaction, depression, and sclerosis.^1^^,^^11^^,^^31^ In the four boys, asymptomatic OCD lesions appeared in the preadolescent capitellum with the skeletal age score of 3 points (0-9 points system), the ossifying subchondral bone surface was still spherical in shape (Figs. 1, *B*, and 2, *B*), and the overlying cartilage remained thicker laterally (Fig. 3, *A*). During a pitch, compressive and shear force from the radial head to the preadolescent capitellum may mainly act as a shear force on the spherical subchondral bone surface in the lateral aspect of the capitellum (Fig. 3, *B*), and accelerated elbow extension increases this shear force. The secondary physis is responsible for the enlargement of the secondary ossification center and has been shown to be the site with weakest resistance to shear force.^7^^,^^19^^,^^30^ Therefore, the most reasonable candidate of repetitive microtrauma leading to subchondral bone rarefaction may be stress injury involving the secondary physis (secondary PSI) of the ossifying subchondral bone surface in the preadolescent capitellum (Fig. 3, *B*). As primary PSIs show the adjacent bone rarefaction, depression, and sclerosis, secondary PSIs may interfere with endochondral ossification at the subchondral bone surface, showing subchondral bone rarefaction, depression, and sclerosis (Fig. 3, *C*). The subchondral bone plate, as seen in adults, has not yet formed in children. Once stress injury occurs through the secondary physis in the preadolescent capitellum, the marrow cells are released to an injured space, resulting in fibrocartilaginous tissue covering the injured surface, accompanied by marrow edema and reactive osteosclerosis.

When APR45 showed rarefaction, flattening, and depression of subchondral bone, CT scan showed small subchondral cysts in the flattened bone (Figs. 1 and 2). The subchondral bone rarefaction may reflect an early phase of subchondral bone cyst formation, and subchondral bone depression with an uneven sclerotic floor may result from side-by-side concurrence of subchondral bone cysts. Subchondral bone flattening-depression progressed and expanded from the lateral to the central aspect of the capitellum (Figs. 1 and 2). Micromovements of the overlying articular fragment may have a role of encouraging the penetration of joint fluid and fibrocartilage into the marrow cavities, leading to subchondral bone cysts and depression.^9^^,^^17^

This study has demonstrated the following progression of early OCD. 1) During repetitive pitches, the secondary physis on the spherical subchondral bone surface in the lateral aspect of the preadolescent capitellum may be affected by increased shear forces (Fig. 3, *A*, *B*), causing stress injury at the secondary physis that is mechanically weakest against shear force (Fig. 3, *B*). 2) The earliest detectable radiographic change in OCD is subtle rarefaction surrounded by sclerosis within a limited area of the ossifying subchondral bone in the lateral aspect of the capitellum (Figs. 1, *B*, and 2, *B*). 3) Repetitive micromovements of the overlying cartilage on the injured surface may encourage side-by side concurrence of subchondral bone cysts (Fig. 3, *D*), leading to subchondral bone depression with an uneven floor surrounded by reactive osteosclerosis (Fig. 3, *D*). Furthermore, secondary PSI may expand from the lateral to the central aspect of the capitellum (Fig. 3, *D*), followed by subchondral bone rarefaction, flattening, and depression (Figs. 1 and 2).

There were several limitations to this study. Among 120 patients with OCD, only 4 cases had consecutive radiographs showing the changes from the intact to OCD. The earliest radiographic changes had not been reported. Use of CT and MRI may possibly have demonstrated features before APR45 revealed subtle changes. However, CT or MRI before radiographic diagnosis of OCD is very rare. The absence of timely CT images in case 4 was regrettable, although they were obtained 13 months after the diagnosis. We had no opportunities to obtain histologic data for the stable lesions.

## Conclusion

This study showed that the earliest detectable radiographic change leading to OCD was subtle rarefaction surrounded by osteosclerosis within a limited area of the ossifying subchondral bone surface in the lateral aspect of the preadolescent capitellum. These features gradually became more evident and expanded, eventually leading to flattening and depression of the subchondral bone, accompanied by subchondral bone cysts. Subchondral bone rarefaction may be secondary to repetitive microtrauma at the ossifying subchondral bone surface (secondary PSI), caused by repetitive forces on the preadolescent capitellum. OCD may silently begin with secondary PSI. Micromovements of the overlying cartilage would allow the concurrent development of subchondral bone cysts, resulting in bone depression with an uneven floor surrounded by osteosclerosis.

## Acknowledgment

The authors sincerely thank Tadanobu Nemoto, MD (Chief Director, Izumi Orthopaedic Hospital, Sendai, Japan), Mikio Harada, MD, PhD, and Masahiro Maruyama, MD, PhD for their kind support.

## Disclaimers:

Funding: No funding was disclosed by the authors.

Conflicts of interest: The authors, their immediate families, and any research foundation with which they are affiliated have not received any financial payments or other benefits from any commercial entity related to the subject of this article.

## References

[1] Arnold A., Thigpen C.A., Beattie P.F., Kissenberth M.J., Shanley E. (2017). Overuse physeal injuries in youth athletes. Sports Health.

[2] Barrie H.J. (1987). Osteochondritis dissecans 1887–1987. A centennial look at König’s memorable phrase. J Bone Joint Surg Br.

[3] Beck A., Murphy D.J., Carey-Smith R., Wood D.J., Zheng M.H. (2016). Treatment of articular cartilage defects with microfracture and autologous matrix-induced chondrogenesis leads to extensive subchondral bone cyst formation in a sheep model. Am J Sports Med.

[4] Chiroff R.T., Cooke C.P. (1975). Osteochondritis dissecans: a histologic and microradiographic analysis of surgically excised lesions. J Trauma.

[5] Diméglio A., Charles Y.P., Daures J.P., de Rosa V., Kaboré B. (2005). Accuracy of the sauvegrain method in determining skeletal age during puberty. J Bone Joint Surg Am.

[6] Fa K., Bauer E., Harland U. (2005). Are bone bruises a possible cause of osteochondritis dissecans of the capitellum? A case report and review of the literature. Arch Orthop Trauma Surg.

[7] Flachsmann R., Broom N.D., Hardy A.E., Moltschaniwskyj G. (2000). Why is the adolescent joint particularly susceptible to osteochondral shear fracture?. Clin Orthop Relat Res.

[8] Funakoshi T., Furushima K., Miyamoto A., Kusano H., Horiuchi Y., Itoh Y. (2019). Predictors of unsuccessful nonoperative management of capitellar osteochondritis dissecans. Am J Sports Med.

[9] Gao L., Cucchiarini M., Madry H. (2020). Cyst formation in the subchondral bone following cartilage repair. Clin Transl Med.

[10] Harada M., Takahara M., Hirayama T., Sasaki J., Mura N., Ogino T. (2012). Outcome of nonoperative treatment for humeral medial epicondylar fragmentation before epiphyseal closure in young baseball players. Am J Sports Med.

[11] Harada M., Takahara M., Maruyama M., Kondo M., Uno T., Takagi M. (2018). Outcome of conservative treatment for little league shoulder in young baseball players: factors related to incomplete return to baseball and recurrence of pain. J Shoulder Elbow Surg.

[12] Harada M., Takahara M., Sasaki J., Mura N., Ito T., Ogino T. (2006). Using sonography for the early detection of elbow injuries among young baseball players. AJR Am J Roentgenol.

[13] Hefti F., Beguiristain J., Krauspe R., M€oller-Madsen B., Riccio V., Tschauner C. (1999). Osteochondritis dissecans: a multicenter study of the European Pediatric Orthopedic Society. J Pediatr Orthop B.

[14] Hidaka S., Sugioka Y., Kameyama H. (1983). Pathogenesis and treatment of osteochondritis dissecans--an experimental study on chondral and osteochondral fractures in adult and young rabbits. Nihon Seikeigeka Gakkai Zasshi.

[15] Iwasaki N., Kamishima T., Kato H., Funakoshi T., Minami A. (2012). A retrospective evaluation of magnetic resonance imaging effectiveness on capitellar osteochondritis dissecans among overhead athletes. Am J Sports Med.

[16] Kajiyama S., Muroi S., Sugaya H., Takahashi N., Matsuki K., Kawai N. (2017). Osteochondritis dissecans of the humeral capitellum in young athletes: comparison between baseball players and gymnasts. Orthop J Sports Med.

[17] Kaspiris A., Hadjimichael A.C., Lianou I., Iliopoulos I.D., Ntourantonis D., Melissaridou D. (2023). Subchondral bone cyst development in osteoarthritis: from pathophysiology to bone microarchitecture changes and clinical implementations. J Clin Med.

[18] Kida Y., Morihara T., Kotoura Y., Hojo T., Tachiiri H., Sukenari T. (2014). Prevalence and clinical characteristics of osteochondritis dissecans of the humeral capitellum among adolescent baseball players. Am J Sports Med.

[19] Kikukawa H., Tomatsu T., Akasaka O., Yasui Y. (1996). Mechanical properties of pig articular bone-cartilage junction. Jpn Soc Clin Biomech Relat Res.

[20] Koch S., Kampen W.U., Laprell H. (1997). Cartilage and bone morphology in osteochondritis dissecans. Knee Surg Sports Traumatol Arthrosc.

[21] König F. (2013). The classic: on loose bodies in the joint. 1887. Clin Orthop Relat Res.

[22] Kusumi T., Ishibashi Y., Tsuda E. (2006). Osteochondritis dissecans of the elbow: histopathological assessment of the articular cartilage and subchondral bone with emphasis on their damage and repair. Pathol Int.

[23] Langenskiöld A. (1955). Can osteochondritis dissecans arise as a sequel of cartilage fracture in early childhood? An experimental study. Acta Chir Scand.

[24] Linden B., Telhag H. (1977). Osteochondritis dissecans. A histologic and autoradiographic study in man. Acta Orthop Scand.

[25] Logli A.L., Bernard C.D., O’Driscoll S.W., Sanchez-Sotelo J., Morrey M.E., Krych A.J. (2019). Osteochondritis dissecans lesions of the capitellum in overhead athletes: a review of current evidence and proposed treatment algorithm. Curr Rev Musculoskelet Med.

[26] Maruyama M., Takahara M., Harada M., Satake H., Takagi M. (2014). Outcomes of an open autologous osteochondral plug graft for capitellar osteochondritis dissecans: time to return to sports. Am J Sports Med.

[27] Maruyama M., Takahara M., Satake H. (2018). Diagnosis and treatment of osteochondritis dissecans of the humeral capitellum. J Orthop Sci.

[28] Matsuura T., Kashiwaguchi S., Iwase T., Takeda Y., Yasui N. (2008). Conservative treatment for osteochondrosis of the humeral capitellum. Am J Sports Med.

[29] Matsuura T., Wada K., Suzue N., Iwame T., Fukuta S., Sairyo K. (2017). Bilateral osteochondritis dissecans of the capitellum in fraternal twins: a case report. JBJS Case Connect.

[30] Moen C.T., Pelker R.R. (1984). Biomechanical and histological correlations in growth plate failure. J Pediatr Orthop.

[31] Myers N.L., Kennedy S.M., Arnold A.J., Gehring Z.A., Kruseman K.J., Conway J.E. (2024). A narrative review of little league shoulder: proximal humeral physis widening is only one piece of the puzzle, it is time to consider posterior glenoid dysplasia. JSES Int.

[32] Nissen C.W. (2014). Osteochondritis dissecans of the elbow. Clin Sports Med.

[33] Niu E.L., Tepolt F.A., Bae D.S., Lebrun D.G., Kocher M.S. (2018). Nonoperative management of stable pediatric osteochondritis dissecans of the capitellum: predictors of treatment success. J Shoulder Elbow Surg.

[34] Olstad K., Shea K.G., Cannamela P.C., Polousky J.D., Ekman S., Ytrehus B. (2018). Juvenile osteochondritis dissecans of the knee is a result of failure of the blood supply to growth cartilage and osteochondrosis. Osteoarthritis Cartilage.

[35] Phillips H.O., Grubb S.A. (1985). Familial multiple osteochondritis dissecans. Report of a kindred. J Bone Joint Surg Am.

[36] Saper M.G., Bompadre V., Schmale G.A., Menashe S., Burton M., Nagle K. (2021). Association between 45 flexion anteroposterior elbow radiographs and diagnostic accuracy of capitellum osteochondritis dissecans. Am J Sports Med.

[37] Satake H., Takahara M., Harada M., Maruyama M. (2013). Preoperative imaging criteria for unstable osteochondritis dissecans of the capitellum. Clin Orthop Relat Res.

[38] Skagen P.S., Horn T., Kruse H.A., Staergaard B., Rapport M.M., Nicolaisen T. (2011). Osteochondritis dissecans (OCD), an endoplasmic reticulum storage disease? A morphological and molecular study of OCD fragments. Scand J Med Sci Sports.

[39] Stevens K.J., Chaudhari A.S., Kuhn K.J. (2024). Differences in anatomic adaptation and injury patterns related to valgus extension overload in overhead throwing athletes. Diagnostics (Basel).

[40] Stougaard J. (1964). Familial occurrence of osteochondritis dissecans. J Bone Joint Surg Br.

[41] Takahara M. (2023). Osteochondritis dissecans of the elbow: recent evolution of pathogenesis, imaging, and treatment modalities. JSES Int.

[42] Takahara M., Maruyama M., Uno T., Harada M., Satake H., Takahara D. (2021). Progression of epiphyseal cartilage and bone pathology in surgically treated cases of osteochondritis dissecans of the elbow. Am J Sports Med.

[43] Takahara M., Mura N., Sasaki J., Harada M., Ogino T. (2007). Classification, treatment, and outcome of osteochondritis dissecans of the humeral capitellum. J Bone Joint Surg Am.

[44] Takahara M., Ogino T., Takagi M., Tsuchida H., Orui H., Nambu T. (2000). Natural progression of osteochondritis dissecans of the humeral capitellum: initial observations. Radiology.

[45] Takahara M., Ogino T., Tsuchida H., Takagi M., Kashiwa H., Nambu T. (2000). Sonographic assessment of osteochondritis dissecans of the humeral capitellum. Am J Roentgenol.

[46] Takahara M., Shundo M., Kondo M., Suzuki K., Nambu T., Ogino T. (1998). Early detection of osteochondritis dissecans of the capitellumin young baseball players. Report of three cases. J Bone Joint Surg Am.

[47] Takahara M., Uno T., Maruyama M., Harada M., Mitachi R., Ono H. (2022). Conservative treatment for stable osteochondritis dissecans of the elbow before epiphyseal closure: effectiveness of elbow immobilization for healing. J Shoulder Elbow Surg.

[48] Takahara M., Uno T., Maruyama M., Harada M., Satake H., Takahara D. (2021). Staging of osteochondritis dissecans of the elbow based on pathological progression in the partially detached articular fragment. J Shoulder Elbow Surg.

[49] Tallqvist G. (1962). The reaction to mechanical trauma in growing articular cartilage. An experimental study on rabbits and a comparison of the results with the pathological anatomy of osteochondritis dissecans. Acta Orthop Scand Suppl.

[50] Uno T., Takahara M., Maruyama M., Harada M., Satake H., Takagi M. (2021). Qualitative and quantitative assessments of radiographic healing of osteochondritis dissecans of the humeral capitellum. JSES Int.

[51] Uozumi H., Sugita T., Aizawa T., Takahashi A., Ohnuma M., Itoi E. (2009). Histologic findings and possible causes of osteochondritis dissecans of the knee. Am J Sports Med.

[52] Yonetani Y., Nakamura N., Natsuume T., Shiozaki Y., Tanaka Y., Horibe S. (2010). Histological evaluation of juvenile osteochondritis dissecans of the knee: a case series. Knee Surg Sports Traumatol Arthrosc.

